# Optimizing gamma irradiation for mutation breeding in seedless barberry (*Berberis vulgaris* L.): Establishing LD_50_ and GR_50_ thresholds

**DOI:** 10.1371/journal.pone.0334218

**Published:** 2025-11-13

**Authors:** Mehri Asadi Zargh Abad, Gholamreza Rabiei, Ali Akbar Ghasemi-Soloklui, Mojtaba Kordrostami, Mohammad Rabiei

**Affiliations:** 1 Department of Horticultural Sciences, Faculty of Agriculture, Shahrekord University, Shahr-e Kord, Iran; 2 Nuclear Agriculture Research School, Nuclear Science and Technology Research Institute (NSTRI), Karaj, Iran; 3 Department of Plant Breeding and Biotechnology, Faculty of Agriculture, Shahrekord University, Shahr-e Kord, Iran; Helwan University, EGYPT

## Abstract

Native to Iran, seedless barberry (*Berberis vulgaris* L.) is a nutritionally and economically important crop prized for its therapeutic uses and food industry applications. Nevertheless, the vegetative propagation of the plant and lack of seeds limit conventional breeding techniques, therefore reducing genetic variation and impeding cultivars improvement. This work sought to maximize gamma radiation dosages to cause mutations in seedless barberry hence increasing genetic variability for breeding projects. Hardwood cuttings were subjected to gamma radiation dosages of 0 (control), 10, 20, 30, 40, 50, 60, 70, 80, 90, and 100 Gy using a Cobalt-60 source. Under both *in vitro* and *in vivo* settings, the impacts on survival rates, leaf and shoot development, and morphometric features were assessed. Results revealed that rising radiation doses significantly reduced survival rates and growth metrics. The LD_50_ (Lethal dose) was determined to be roughly 19 Gy for the in vivo and 13.6 Gy for the in vitro. Gamma irradiation negatively influenced plant growth according to values for leaf length, leaf width, fresh weight, and dry weight growth reduction (GR_50_). Optimal dosages for causing mutations, while preserving survival, were found to be 15 Gy for the in vivo and 10 Gy for the in vitro.

## Introduction

Seedless barberry (*Berberis vulgaris* L. *var. asperma*.), belonging to the Berberidaceae family, is a deciduous shrub native to Iran and holds substantial economic, nutritional, and medicinal significance both locally and globally. Renowned for its vibrant red and tart berries, seedless barberry is rich in bioactive compounds such as berberine and berberamine, which possess potent antioxidant properties and have been utilized in traditional medicine for their antimicrobial and anti-inflammatory effects [1]. The fruits are widely used in the food industry as natural flavoring agents and colorants, enhancing the nutritional value and sensory attributes of various culinary products. Moreover, the global demand for natural health-promoting ingredients has spurred interest in seedless barberry as a valuable crop with significant export potential [2].

Despite its importance, the cultivation and genetic improvement of seedless barberry face considerable challenges due to its reproductive biology. Due to its seedless the plant propagates vegetatively through root suckers, resulting in limited genetic diversity [3]. This lack of genetic variation restricts the potential for traditional breeding methods that rely on sexual reproduction and seed formation, hindering the development of new varieties with enhanced traits such as higher yield, improved stress tolerance, and superior phytochemical profiles. The lack of genetic variability of vegetatively propagated plants also increases their vulnerability to diseases, pests, and environmental stresses, posing a threat to sustainable production and agricultural profitability [4].

Addressing these breeding challenges necessitates alternative strategies to introduce genetic variation into seedless barberry populations. Induced mutation breeding emerges as a promising approach to overcome the limitations posed by the plant’s reproductive constraints. Physical mutagens like gamma radiation can induce genetic mutations at the DNA level, leading to new genotypes with potentially beneficial traits without the need for sexual reproduction [5]. Mutation breeding has been successfully applied in various crops to enhance desirable characteristics, contributing significantly to food security and agricultural sustainability [6].

In the breeding of plants, gamma irradiation clearly offers benefits over chemical mutagenesis, particularly for crops like seedless barberry. Gamma irradiation can enter plant tissues more deeply compared to chemical agents like Ethyl methyl sulphonate (EMS), which mostly produce minor mutation and specific changes in genes because chemical mutagens induce point mutations [7]. This can result in bigger rearrangements and provide much-needed genetic variability for species with minimal variation, such as seedless barberry [8]. Additionally, it results in more reliable mutation effects, which facilitates the selection of advantageous features. Modern methods, such as CRISPR-Cas, are accurate, but they only target specific genes and are often limited by incomplete genomic information [9,10]. Gamma irradiation, on the other hand, has a long history of effectiveness in mutation breeding and causes a variety of mutations that increase genetic diversity.

Optimizing gamma radiation doses is crucial for inducing beneficial mutations while minimizing detrimental effects on plant growth and survival. Determining the lethal dose (LD_50_) and growth reduction doses (GR_25_, GR_50_, GR_75_) provide valuable insights into the plant’s radiosensitivity and guides the selection of appropriate radiation levels for breeding programs [11]. Such advancements have the potential to significantly impact the agricultural sector by improving crop performance, offering economic benefits to farmers, and contributing to the global supply of nutritionally rich and health-promoting foods.

To date, there is a conspicuous lack of research on inducing mutations in seedless barberry using gamma radiation, highlighting a critical gap in the knowledge and application of genetic breeding through mutation techniques in this species. This study pioneers the exploration of gamma radiation as a tool for mutation induction in seedless barberry, with the aim of optimizing radiation doses that balance effective mutagenesis and plant viability. By identifying the optimal gamma radiation doses, we can accelerate mutation breeding programs, facilitating the development of superior seedless barberry cultivars with enhanced yield, quality, and stress resistance. Thus, the primary objectives of this investigation were to: a) Assess how different gamma radiation doses affected the morphometric characteristics, growth, and survival of seedless barberry in both in vitro and in vivo conditions. b) Determine the optimum gamma radiation doses based on growth reduction metrics (GR_25_, GR_50_, and GR_75_) so as to help future breeding programs for seedless barberry improvement by inducing advantageous mutations while maintaining plant viability, in vitro and in vivo conditions.

## Materials and methods

### Plant materials

One-year-old hardwood cuttings of seedless barberry (*Berberis vulgaris* L. var. asperma) were collected during the dormant season from a single, healthy, elite mother plant grown in a commercial orchard in South Khorasan Province (Birjand), Iran. This mother plant was previously identified for its superior fruit quality and has been clonally maintained to ensure genetic uniformity. All cuttings used in the experiment were genetically identical to minimize background genetic variation. This ensured that any changes observed after irradiation could be attributed to mutagenic effects rather than pre-existing variability. Cuttings were selected based on uniform size (approximately 15 cm in length and 0.5 cm in diameter) and absence of any visible diseases or pest infestation. To satisfy the chilling requirement and promote uniform bud break, the cuttings were stored at 4°C in a cold chamber for 45 days prior to treatment.

### Gamma irradiation

The irradiation of cuttings was performed at the Nuclear Science and Technology Research Institute in Karaj, Iran, using a Cobalt-60 (Co-60) gamma radiation source. Dosimetry calibration was performed to deliver a precise dose rate of 1 Gy/min. Cuttings were divided into eleven treatment groups (200 cuttings per treatment), including a non-irradiated control (0 Gy) and ten irradiation treatments at doses of 10, 20, 30, 40, 50, 60, 70, 80, 90, and 100 Gy. Exposure times were adjusted accordingly to deliver the exact dosage required. Calibration procedures were conducted prior to irradiation to verify dose accuracy.

### In vivo culture condition

After irradiation, the basal ends of the cuttings were treated with indole-3-butyric acid (IBA) to promote rooting. The cuttings were dipped in a 2000 mg L^−1^ indole-3-butyric acid (IBA) solution for 5 seconds. Control cuttings were similarly treated without irradiation. The cuttings were then planted in plastic pots (20 × 25 cm) containing washed and sterilized sand as the rooting medium.

The pots were placed in a greenhouse and temperature maintained 25 ± 2°C during the day and 18 ± 2°C at night, with a relative humidity of 70–80%. A bottom heating system set at 25°C was used to enhance rooting. The greenhouse provided a 16-hour photoperiod using fluorescent lamps that delivered a light intensity of approximately 200 µmol m^−2^s^−1^ at canopy level. Supplemental lighting provided a 16-hour photoperiod with approximately 200 µmol m⁻^2^s⁻^1^ of light intensity. Regular irrigation was performed to keep the sand medium moist but not waterlogged.

### In vitro culture condition

Cuttings assigned to in vitro treatment were first surface sterilized. After initial washing under running tap water with detergent for 30 minutes, the cuttings were sterilized using 70% ethanol for 60 seconds, followed by 15 minutes in a 15% (v/v) sodium hypochlorite solution with Tween-20. After three rinses with sterile distilled water, explants were cultured on Murashige and Skoog (MS) basal medium containing 30 g L^−1^ sucrose and solidified with 7 g L^−1^ agar. The pH was adjusted to 5.8 before autoclaving. No plant growth regulators were added. Cultures were maintained at 25 ± 2°C under a 16-hour photoperiod (40 µmol m⁻^2^s⁻^1^).

### Experimental design and statistical analysis

The experiment was conducted using a completely randomized design, comprising five replicates per treatment under in vivo conditions and three replicates per treatment under in vitro conditions. In vitro, each replicate consisted of four microcuttings with two buds; in vivo, each replicate consisted of ten cuttings. Plant survival, number of buds, leaf dimensions (length and width), and biomass (fresh and dry weights) were measured one-month post-treatment. Significant differences between treatment means were determined using LSD (Least Significant Difference) test at the 0.05 probability level.

Data were subjected to ANOVA using SPSS statistical software (version 25.0; IBM Corp., Armonk, NY, USA). Prior to ANOVA, data were tested for normality using the Shapiro-Wilk test and for homogeneity of variances using Levene’s test. When necessary, data were transformed (e.g., square root or logarithmic transformation) to meet the assumptions of ANOVA. ANOVA was conducted to assess the effects of gamma radiation doses on the measured traits. Significant differences among treatment means were determined using LSD at the 0.05 probability level.

Regression analyses were performed to model the relationship between gamma radiation doses and survival rates, as well as growth traits. The lethal dose (LD_50_) and growth reduction doses (GR_25_, GR_50_, GR_75_) were estimated using Ghasemi-Soloklui [11] and dose-response curves generated with GraphPad Prism software (version 9.0; GraphPad Software, San Diego, CA, USA). Confidence intervals at 95% were calculated for LD_50_ and GR_50_ estimates.

After confirming there were no significant differences between the repeats, the data were combined for analysis. We assessed the precision of the experiments using coefficients of variation (CV). Each treatment had a total of eight repetitions, with four repetitions per experiment, which is reflected in the tables where the means were calculated.

### Plant growth trait measurements

In both conditions (In vivo and in vitro culture) one month after planting, the survival percentage was calculated by dividing the number of surviving cuttings by the total number of cuttings per treatment and multiplying by 100. Morphological traits measured included number of buds, leaf length (mm), and leaf width (mm). The fresh weight of both shoots and leaves was measured as fresh weight using a digital analytical balance with an accuracy of 0.001 g. For dry weight determination, the samples, consisting of both leaves and shoots, were oven-dried at 65°C for 72 hours until a constant weight was achieved.

## Results

### Effect of mutagenesis on hardwood cutting survival percentage

The response of seedless barberry to gamma irradiation in the greenhouse exhibited significant differences in survival rates (P < 0.01) across various treatments. The highest survival rate in vivo (99.58%) was recorded in the control group, while the lowest survival rate (17.29%) occurred at 40 Gy. Survival rates at 10 Gy and 20 Gy were 60.6 and 43.2%, respectively, while no survival was recorded at doses of 50 Gy or higher under in vivo conditions. These results indicate a significant dose-dependent reduction in survival, highlighting the strong impact of gamma radiation on plant viability.

No survival was observed in cuttings exposed to 50 Gy or higher under in vivo conditions. Overall, hardwood cuttings demonstrated better survival rates at low to moderate gamma-ray doses compared to the highest dose. The findings indicate that mutagenesis had a considerable impact on survival in vivo.

The explants experiment in vitro also showed significant differences in survival rates (P < 0.01) between the control, 10 Gy (58.2%), and 20 Gy (25%) treatments. The control group exhibited the highest survival rate (95.83%), with no survival observed at 30 Gy or higher. Additionally, a progressive decline in survival percentage was noted with increasing gamma irradiation doses (Table 1). The observed survival rates in both tissue culture and field conditions suggest that gamma radiation has a more pronounced mutagenic effect on younger tissues, such as those used in in vitro culture.

**Table 1:** The effect of gamma irradiation doses on survival rates of hardwood cuttings of barberry (*Berberis vulgaris*) in vivo and in vitro conditions.

	Gamma dose (Gy)	Number of cuttings	Survival (%)	Survival over control (%)	Reduction over control (%)
In vivo	0	30	99.58	100	–
	10	30	60.65	61.39	38.61
	20	30	43.29	43.81	56.19
	30	30	17.29	17.5	82.5
	40	30	17.29	17.5	82.5
CV (%)			6.7	6.8	6.1
In vitro	Gamma dose (Gy)	Number of cuttings	Survival (%)	Survival over control (%)	Reduction over control (%)
	0	24	95.83	100	–
	10	24	58.33	59.04	40.96
	20	24	25	25.30	74.70
	30	24	0	0	100
	40	24	0	0	100
CV (%)			10.2	8.1	9.3

Values are means ± SE. Within each column, means followed by the same letter are not significantly different according to Fisher’s LSD test at P < 0.05.

**Fig 1:**
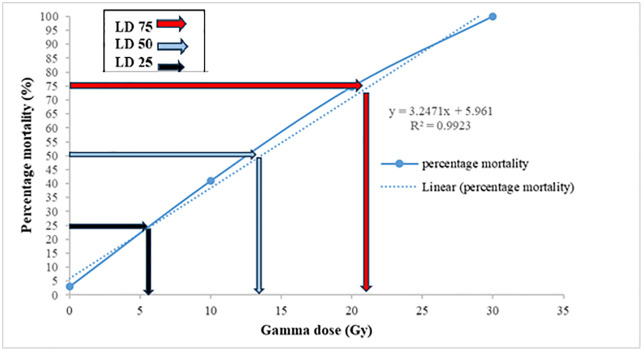
Survival percentage of seedless barberry cuttings under in vivo conditions at different gamma irradiation doses. Values represent means ± SE.

**Fig 2:**
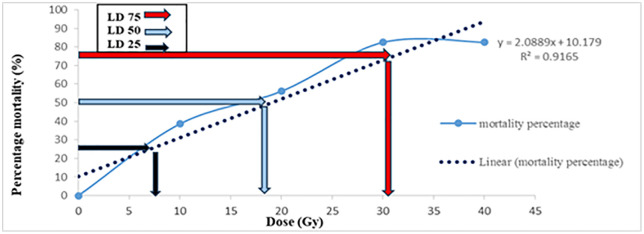
Survival percentage of seedless barberry under in vitro culture across gamma irradiation doses. Values represent means ± SE. Complete mortality was observed at doses ≥30 Gy.

Figs 1 and 2 illustrate the lethal dose analysis for in vivo and in vitro conditions, respectively, highlighting the distinct thresholds where survival starts declining sharply.

In vivo survival began to decline noticeably at doses above 10 Gy, with a significant reduction observed beyond 20 Gy, and complete mortality occurring at 50 Gy (Fig 1). In contrast, in vitro conditions exhibited a sharper and earlier decline in survival, with significant decreases starting at 10 Gy and complete mortality reached at 30 Gy (Fig 2). These results indicate that in vitro culture is more sensitive to gamma radiation than in vivo conditions, likely due to the greater vulnerability of tissue cultures to environmental stressors and the absence of buffering mechanisms present in soil-based systems. The highest survival rates in both systems were observed at doses below 10 Gy.

These findings indicate that gamma irradiation significantly reduces survival in both in vivo and in vitro conditions, with a steep decline observed beyond specific threshold doses. It suggests that seedless barberry cuttings exhibit differential sensitivity to radiation, depending on the applied dose. The consistent decline in survival with increasing radiation points to the vulnerability of hardwood cuttings and tissue culture to radiation-induced stress.

### Determining the LD_25_, LD_50_, and LD_75_ of mutagens

The linear regression model based on radiation dose was Y = 2.0889x + 10.179 with an R^2^ value of 0.9165 (Fig 1). According to the findings, LD_25_ values for cuttings in the greenhouse were 7, 19 Gy for LD_50_, and 31 Gy for LD_75_ (Fig 1). Therefore, gamma radiation doses between 12–21 Gy in the in vivo culture in *Berberis vulgaris* can penetrate in bud meristem and affect survival rate of the plant. Additionally, in vitro conditions showed an LD_50_ value of 13.6 Gy for shoots (Fig 2). According to the findings, the LD_25_ and LD_75_ values for cuttings under in vitro conditions were estimated at 6 and 22 Gy, respectively (Fig 2).

These LD values demonstrate the radiation tolerance of seedless barberry and provide insights into effective dose levels for penetration of gamma rays in bud meristem. The high R^2^ value (0.91 in vivo and 0.99 in vitro) of the regression model suggests a strong correlation between gamma irradiation dose and survival, indicating the robustness of the model in predicting radiation-induced responses.

### Effects of mutagens on leaf traits in vivo and in vitro

The analysis of variance results (Tables 1 and 2 in the Appendix) confirm that gamma irradiation significantly (P < 0.01) affected all evaluated morphometric traits of seedless barberry under both in vivo and in vitro conditions. In the greenhouse (in vivo), increasing radiation doses led to a notable decrease in the number of buds, leaf dimensions, and biomass (fresh and dry weight). Similarly, in the in vitro culture, a dose-dependent reduction in growth parameters was observed. These statistical findings are in line with the trends described earlier, including the reductions in survival and growth at higher doses, and they emphasize the strong mutagenic impact of gamma rays on plant morphology and biomass production.

**Table 2:** Average effects of gamma irradiation doses on morphometric traits of barberry (*Berberis vulgaris*) in vivo and in vitro conditions.

	Gamma dose (Gy)	Number of buds	Leaf length (mm)	Leaf width (mm)	Fresh weight of leaf and shoot (mg)	Dry weight of leaf and shoot (mg)
In vivo	0	12 ± 0.2 a	15 ± 0 a	6.7 ± 0.3 a	353.8 ± 0.3 a	74.6 ± 0.3 a
	10	10.4 ± 0.5ab	8.8 ± 0.3 b	3.9 ± 0.2 b	240.6 ± 0.6 b	41.7 ± 0.2 b
	20	10 ± 0.1ab	2.4 ± 0.4 c	2 ± 0.2 c	83.64 ± 0.3 c	15.6 ± 0.3 c
	30	8 ± 0.4 b	0.4 ± 0.05 d	1.2 ± 0.01 d	18.4 ± 0.2 d	3.5 ± 0.01 d
CV (%)		8.4	9.3	9.5	10.2	7.8
In vitro	0	8.8 ± 0 a	10.6 ± 0.3 a	4 ± 0.5 a	58.6 ± 1.7 a	5.8 ± 1.2 a
	10	5.4 ± 0.6 b	7.4 ± 0.9 ab	3 ± 0.5 a	23.6 ± 1.4 ab	2.1 ± 0.2 b
	20	2.4 ± 0 c	6 ± .0.6 b	0.2 ± 0.002 b	10.3 ± 1.2 b	1.6 ± 0.6 c
CV (%)		6.9	9.3	3.01	3.7	8.8

Within each sub-section (in vivo or in vitro), means in the same column followed by different letters are significantly different based on Fisher’s LSD test (P < 0.05).

The effects of gamma radiation on barberry leaf characteristics in the greenhouse are summarized in Table 2. In vivo Significant differences in leaf width and length were observed as a result of gamma radiation exposure (Table 2). The control group exhibited maximum leaf length (15 mm) and width (6.7 mm), while the smallest leaf dimensions were recorded at 30 Gy, with a leaf length of 0.4 mm and a width of 1.2 mm (Table 2). Leaf length and width consistently decreased with increasing radiation doses, showing significant differences among doses of 10, 20, and 30 Gy.

At radiation doses of 10, 20, and 30 Gy, leaf lengths were recorded as 8.8 mm, 2.4 mm, and 0.4 mm, respectively. Correspondingly, leaf widths measured 3.9 mm, 2.0 mm, and 1.2 mm at the same doses.

In vivo Fresh weight also significantly decreased compared to the control (353.8 mg), with weights of 240.6 mg, 83.6 mg, and 18.4 mg observed at doses of 10, 20, and 30 Gy, respectively. Notably, there was a significant difference in dry weight between the 10 Gy (41.7 mg) and 20 Gy (15.6 mg) doses. All gamma radiation doses led to a significant reduction in fresh and dry weight compared to the control (Table 2).

The number of sprouts in vivo condition seedless barberry cuttings decreased with increasing radiation dose, with a significant reduction from the control treatment (12 sprouts) to 30 Gy (8.6 sprouts).

In the in vitro conditions, leaf width and length showed significant differences between the control and gamma radiation doses (10–100 Gy). However, shoots were lost after exposure to 20 Gy in vitro. Leaf length decreased from 10.6 mm in the control to 7.3 and 6.1 mm at 10 and 20 Gy, respectively (Table 2). Additionally, radiation intensity led to a significant decrease in average leaf width, with 20 Gy resulting in a width of 0.2 mm, significantly different from the control (4 mm) (Table 2). The increase in gamma radiation dose resulted in a reduction in fresh leaf weight from 58.6 mg in the control to 10.3 mg at 20 Gy. Dry weight analysis revealed a decrease from 2.1 mg at 10 Gy to 1.6 mg at 20 Gy, showing a significant difference (Table 2). The number of sprouts also showed a decreasing trend under radiation doses, with a significant difference between the control treatment (8 sprouts) and the 10 Gy (4.6 sprouts) and 20 Gy (2 sprouts) treatments.

**Fig 3:**
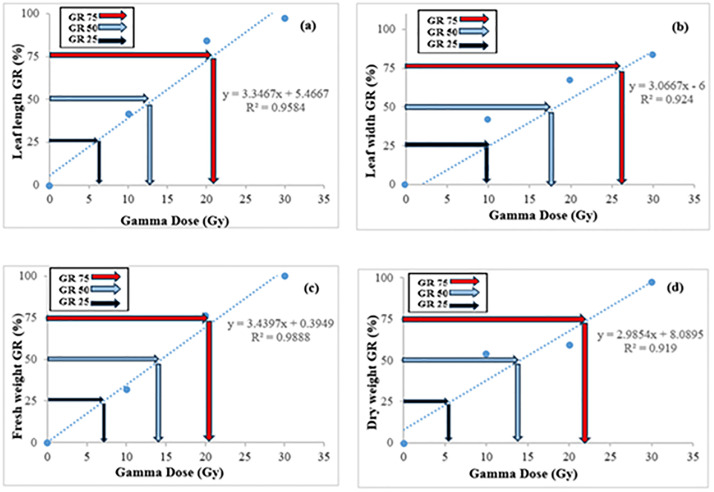
Effect of gamma irradiation on leaf length (a), leaf width (b), fresh weight (c) and dry weight d) in hardwood cuttings of barberry under in vivo conditions. Dose–response curves were fitted using non-linear regression (R^2^ ≥ 0.90). Dotted lines indicate estimated GR₅₀ values.

**Fig 4:**
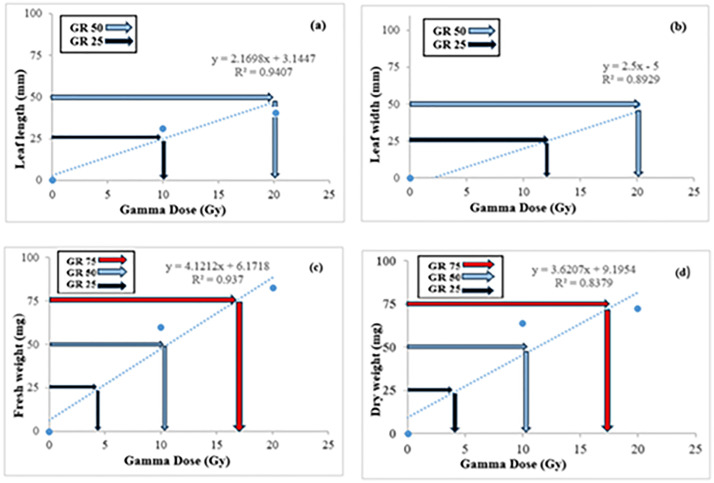
Effect of gamma irradiation on leaf length (a), leaf width (b), fresh weight (c) and dry weight (d) in hardwood cuttings of seedless barberry grown in vitro. Data points represent means ± SE; curves show fitted GR dose-response models.

Figs 3 and 4 provide a visualization of the impact of gamma irradiation on leaf characteristics, such as leaf width, length, fresh weight, and dry weight for in vivo and in vitro conditions, respectively. The decrease in growth characteristics across increasing doses aligns with data from Table 2. Notably, the reduction in leaf length and width at higher doses (30 Gy and above) is especially pronounced, emphasizing the severe effect of higher gamma radiation on vegetative growth.

The greatest leaf length was observed at a dose of 6 Gy (Fig 3a). The effects of gamma radiation on reducing leaf length became apparent at doses above 10 Gy, with a 50% reduction in vegetative growth recorded at 12 Gy and more than 75% at 22 Gy (Fig 3a). The maximum leaf width was recorded at a dose of 10 Gy (Fig 3b), while reductions in leaf width were observed at doses above 10 Gy, with a 50% decrease at 12 Gy and more than 75% at 26 Gy (Fig 3b). Regarding fresh weight, a declining trend began at doses above 10 Gy, with a 50% reduction observed at 18 Gy. The highest fresh weight was recorded at a dose of 7 Gy (Fig 3c). For dry weight, the maximum value was observed at 5.5 Gy, whereas more than 75% reduction occurred at 22 Gy (Fig 3d).

The maximum leaf length was observed at a dose of 10 Gy (Fig 4a), and reductions in leaf length were noted at doses above 10 Gy. A 50% decrease in vegetative growth was observed at 20 Gy (Fig 4a). The greatest leaf width was recorded at 12 Gy (Fig 4b), while reductions began at doses above 15 Gy, with a 50% decrease occurring at 20 Gy (Fig 4b). A declining trend in fresh weight was observed at doses higher than 10 Gy, with a 50% reduction at 10.2 Gy. The maximum fresh weight occurred at 4 Gy (Fig 4c). As for dry weight, the maximum was recorded at 4.5 Gy, while more than 75% reduction was observed at 17 Gy (Fig 4d).

These data highlight the adverse effects of gamma irradiation on vegetative growth parameters, such as leaf size, fresh and dry weight, and number of sprouts. Leaf length, width, and biomass (both fresh and dry weight) were particularly affected, with significant reductions across all treatments compared to the control. The figures support the idea that there are slight differences in radiation sensitivity between in vivo and in vitro conditions, with in vitro samples generally showing this reduction at lower doses compared to in vivo. These findings underline the sensitivity of leaf morphometric traits to radiation and demonstrate the potential use of gamma rays in inducing changes for breeding purposes.

### Sensitivity test to radiation

**Fig 5:**
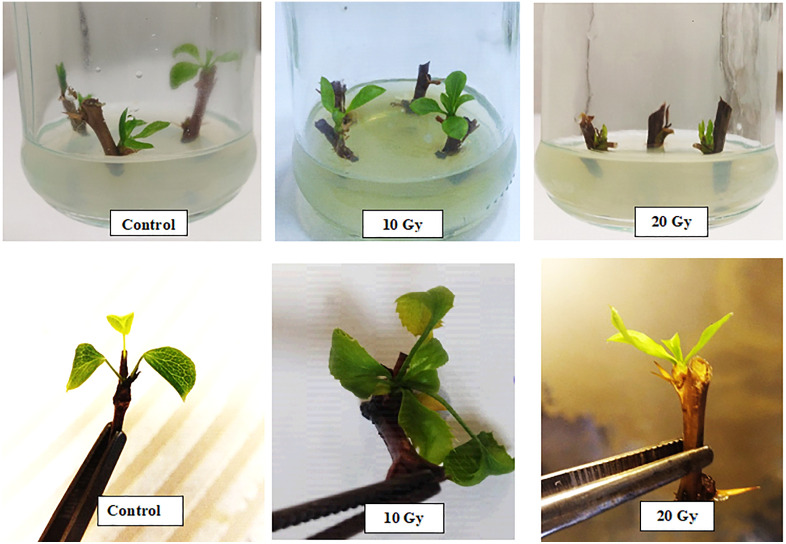
Effects of different gamma irradiation doses on morphometric traits seedless barberry in vitro condition.

The growth reduction (GR) in seedless barberry cuttings, both in vivo and under tissue culture conditions, was assessed using the regression equations derived from gamma irradiation data. Growth reduction for 25% (GR_25_), 50% (GR_50_), and 75% (GR_75_) was calculated for leaf length, leaf width, fresh weight, and dry weight (Figs 3 and 4). The determination coefficients (R^2^) for leaf traits in vitro ranged from 0.83 to 0.94, while those for leaf and shoot characteristics in vivo ranged from 0.91 to 0.98 (Figs 4 and 5). The results indicated that increasing gamma irradiation doses negatively affected barberry growth. Specifically, GR_25_ values for leaf length, leaf width, fresh weight, and dry weight in the in vivo condition were 6, 9.5, 7, and 5.5 Gy, respectively (Fig 3a–d). GR_50_ values for leaf length, leaf width, fresh weight, and dry weight in the in vivo condition were 12, 18, and 14 Gy, respectively. For GR_75_, the doses affecting all shoot characteristics in the in vivo condition of seedless barberry ranged from 20 to 25 Gy (Fig 4a–d). In tissue culture conditions, GR_25_ values for leaf length, fresh weight, and dry weight were 10, 4.5, and 4.5 Gy, while leaf width had a GR_25_ value of 12 Gy (Fig 4a–d). Additionally, the GR_50_ values in vitro were 20 Gy for leaf length and leaf width, and 10.2 Gy for fresh and dry weight, while the GR_75_ values for fresh and dry weight were 17 Gy. (Fig 4a–d).

Figs 3 and 4 show that, while both in vivo and in vitro conditions demonstrate a decline in vegetative growth characteristics with increasing gamma radiation, in vitro samples generally showed this reduction at lower doses compared to in vivo. This difference may be attributed to the protective microenvironment of in vivo plants that helps mitigate some radiation-induced stress.

**Fig 6:**
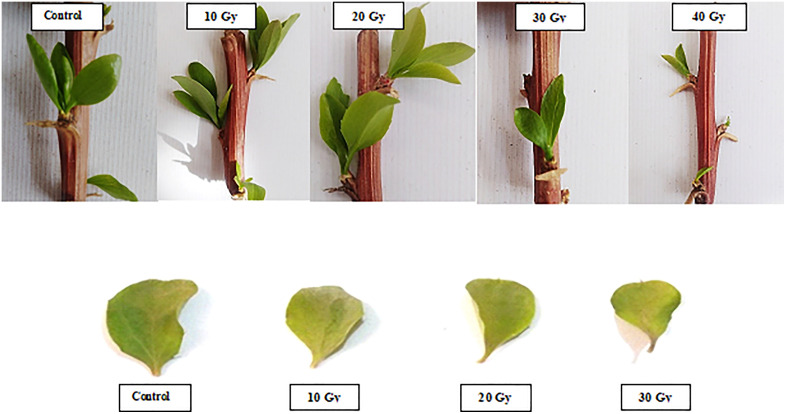
Effects of different gamma irradiation doses on morphometric traits seedless barberry in vivo condition.

These findings indicate that gamma irradiation is effective in significantly reducing growth parameters in barberry (Figs 5 and 6), with radiation intensity being a crucial factor in determining the extent of growth reduction. The GR_25_, GR_50_, and GR_75_ values provide important benchmarks for understanding the sensitivity of different vegetative traits to radiation. The high determination coefficients (R^2^) values suggest that the models used to assess growth reduction are reliable and provide strong predictive power for evaluating the impact of gamma irradiation.

The results demonstrated that gamma irradiation has a significant negative impact on the survival, growth, and morphological traits of barberry hardwood cuttings (Figs 5 and 6). The survival rate decreased drastically with increasing gamma doses, with complete mortality observed at 50 Gy and above for in vivo cultures and at 30 Gy and above for in vitro cultures. The morphometric traits, including the number of buds, leaf length, leaf width, and biomass (fresh and dry weight), also showed significant reductions at higher doses, as summarized in Table 2.

Figs 1–4 provide a visual representation of these findings, illustrating the decrease in survival and growth parameters with increasing gamma doses. The lethal dose (LD_50_) was estimated to be between 10 and 20 Gy for both in vivo and in vitro conditions, indicating the high sensitivity of barberry to radiation. Growth reduction values (GR_25_, GR_50_, and GR_75_) for various traits provide additional insight into the extent of growth inhibition caused by gamma irradiation.

The consistent decrease in survival, growth, and biomass with increasing gamma irradiation doses highlights the potential of gamma rays as a tool for studying radiation-induced stress and its effects on plant growth. However, the significant negative impact on plant health and development suggests that careful consideration must be given to the dose levels when using gamma irradiation for plant breeding or other purposes. The results suggested that gamma irradiation, while effective for radiation-induced phenotypic effects, has the potential to severely hinder vegetative growth and reduce plant survival at relatively low doses. Generally, suitable doses for inducing mutations while maintaining survival based on GR and LD indices, were determined to be 15 Gy in vivo and 10 Gy in vitro.

## Discussion

The present study provided significant insights into the effects of gamma irradiation on the survival, growth, and morphological traits of seedless barberry (*Berberis vulgaris*) cuttings under both in vivo and in vitro conditions. The results demonstrated a clear inverse relationship between gamma radiation doses and plant viability, with higher doses leading to substantial reductions in survival rates and growth parameters [12]. This correlation highlights the sensitivity of seedless barberry to ionizing radiation and underscores the importance of determining optimal irradiation doses for mutation breeding purposes [11].

The decrease in survival rates and morphological traits with increasing gamma radiation doses can be attributed to the deleterious effects of ionizing radiation at the cellular and molecular levels. Gamma rays cause direct damage to DNA by inducing single and double-strand breaks, base modifications, and chromosomal aberrations [13,14]. Such genetic lesions disrupt vital cellular processes like DNA replication and transcription, ultimately leading to cell cycle arrest or programmed cell death if not adequately repaired. Gamma irradiation generates reactive oxygen species (ROS), such as superoxide radicals and hydrogen peroxide, which can damage vital cellular components like proteins, lipids, and DNA [15]. This damage disrupts key processes necessary for growth, leading to inhibited cell division and elongation, which ultimately affects leaf size and biomass [16]. The sensitivity of leaves to such stress may be particularly pronounced due to their active metabolic processes. In vitro conditions might heighten these effects because they lack the natural buffering mechanisms present in vivo, such as soil, which can moderate oxidative damage, or exogenous hormones like rooting compounds that promote recovery. This makes the in vitro environment more susceptible to stress caused by irradiation.

Additionally, gamma irradiation can disturb the balance of plant hormones, which play critical roles in regulating growth. For example, reductions in auxins, such as indole-3-acetic acid (IAA), can impair processes like shoot elongation and leaf expansion [17]. Similarly, lower cytokinin levels, essential for cell division, may contribute to reduced growth. These hormones regulate various aspects of plant growth and development, including cell division, elongation, and differentiation. Alterations in hormonal levels lead to reduced cell proliferation and expansion, manifesting as stunted growth and developmental abnormalities. For instance, high doses of gamma radiation may inhibit the synthesis of indole-3-acetic acid (IAA), a crucial auxin derived from tryptophan, affecting processes like apical dominance and organogenesis [18]. In summary, the reductions in leaf dimensions and biomass can be linked to oxidative stress and disrupted hormonal regulation caused by gamma radiation, with the in vitro environment amplifying these effects. Understanding these mechanisms highlights the importance of carefully optimizing radiation doses to balance mutation induction and plant health.

The comparison of our findings with the previous studies reveals both similarities and species-specific differences in responses to gamma irradiation. Cimen, Yesiloglu [19] reported a 65% survival rate at 60 Gy in sweet orange varieties, indicating greater resistance to gamma irradiation than seedless barberry. These differences underscore the importance of considering species-specific sensitivity when determining optimal irradiation doses for mutation breeding.

The varying sensitivity among species may be attributed to factors such as genome size, DNA repair capacity, antioxidant enzyme activities, and inherent stress tolerance mechanisms [20]. Plants with larger genomes or more efficient DNA repair systems may better withstand radiation-induced damage. Additionally, the physiological state of the plant material, including moisture content and developmental stage, can influence radiosensitivity [21]. Although seedless barberry showed a high degree of sensitivity to gamma irradiation in this study (LD_50_: ~ 19 Gy in vivo; 13.6 Gy in vitro), other plant species, such as citrus (Cimen et al., 2021) and grapevine (Ghasemi-Soloklui et al., 2023), have shown different tolerance levels, highlighting the species-dependent nature of radiosensitivity. Thus, seedless barberry’s higher sensitivity to gamma radiation necessitates careful calibration of irradiation doses to induce mutations without causing excessive mortality or growth inhibition.

Determining the optimal gamma radiation dose is crucial for developing an effective mutation breeding protocol for seedless barberry. The LD_50_ and GR_50_ values identified in this study provide benchmarks for selecting irradiation levels that balance mutation induction with plant viability. By irradiating plant material at doses around the GR_50_ (e.g., 15 Gy in vivo and 10 Gy in vitro), it is possible to generate mutant populations with sufficient genetic variability while maintaining acceptable survival rates [11]. However, this study has limitations that warrant consideration. The evaluation of gamma irradiation effects was confined to early growth stages and did not encompass long-term assessments of plant performance, reproductive capacity, or fruit quality. Mutations may have delayed expressions or impact traits that manifest later in the plant’s life cycle. Additionally, molecular analyses to characterize the genetic changes induced by gamma radiation were not conducted. Such analyses could provide insights into the specific mutations and genetic pathways affected, enhancing the understanding of mutagenesis mechanisms in seedless barberry.

The greater sensitivity of in vitro cultures to gamma irradiation, as shown in Figs 1 and 2, can be explained by several factors: 1) Limited buffering capacity in in vitro media: Unlike soil or natural environments, in vitro media have a limited ability to stabilize pH or counteract chemical changes induced by radiation. This reduced buffering capacity makes in vitro cultures more vulnerable to the harmful effects of gamma rays. 2) Absence of rooting compounds: In vitro cultures often lack rooting hormones or growth regulators, such as auxins, that support plant recovery and stress tolerance. Without these compounds, the ability of the plants to regenerate and cope with radiation damage is significantly reduced. 3) Increased oxidative stress: Gamma radiation generates reactive oxygen species (ROS), causing damage to cellular components like DNA, proteins, and lipids. In vitro systems, being isolated and lacking the natural protective mechanisms present in plants grown in soil, are more susceptible to oxidative stress caused by these ROS. The potential for selecting mutants with advantageous traits holds significant promise for the genetic improvement of seedless barberry. Induced mutations can introduce novel genetic variations that are not readily available through conventional breeding methods, especially in vegetatively propagated species where sexual reproduction is absent [5]. By expanding the genetic diversity of seedless barberry, mutation breeding can facilitate the development of new cultivars with enhanced agronomic performance and nutritional value, contributing to food security and economic benefits for growers. Another critical consideration in vegetative mutagenesis is the potential formation of chimeras. Gamma irradiation may induce mutations in specific cell layers of the shoot apical meristem (e.g., L1, L2, or L3), resulting in periclinal, mericlinal, or sectorial chimeras. These chimeric tissues can exhibit phenotypic changes that are not uniformly expressed throughout the plant and may be lost during subsequent vegetative propagation if non-mutated cells dominate new growth. This phenomenon poses a challenge in mutation breeding of clonally propagated species, as it can obscure the identification of stable mutants. Therefore, while this study reports radiation-induced morphological variation in M1V1 plants, we acknowledge that further propagation (M1V2 and beyond) is necessary to determine whether these changes are genetically fixed. Future studies will incorporate molecular marker analysis and multi-generational tracking to identify and confirm stable, heritable mutations in selected lines.

## Conclusion

Our study demonstrates that based on GR and LD indices, it was shown that 15 Gy in vivo and 10 Gy in vitro were suitable doses for causing mutations while preserving survival. Thus, according to this study, gamma irradiation can effectively induce morphological changes in the M1V1 generation of seedless barberry, contributing to mutation breeding and enhancing morphological variation. Growth and viability were significantly harmed by higher radiation doses, highlighting the significance of cautious dose selection.

In the future, more investigation is required to determine the genetic basis of produced mutations and evaluate their long-term stability. This will optimize the advantages of mutant breeding for crop enhancement and sustainable agriculture.

## Supporting information

S1 File Analysis of variance of the effect of gamma irradiation doses on morphometric traits of seedless barberry (*Berberis vulgaris* L.) under in vivo and in vitro conditions, including tests of normality (Shapiro–Wilk) and homogeneity of variance (Levene). (DOCX)

S2 File Average effects of gamma irradiation doses on morphometric traits of barberry (*Berberis vulgaris* L.) in vivo and in vitro conditions. (XLSX)
