# Supplementary material for: Optimizing gamma irradiation for mutation breeding in seedless barberry (Berberis vulgaris L.): Establishing LD50 and GR50 thresholds
Source: PLoS One. 2025 Nov 13;20(11):e0334218. doi: 10.1371/journal.pone.0334218 (PMC12614536; doi:10.1371/journal.pone.0334218)
Supplement: S1 File — (DOCX) [file pone.0334218.s001.docx]

Table 1. Analysis of variance of the effect of gamma irradiation doses on morphometric traits of seedless barberry (*Berberis vulgaris* L.) under in vivo condition, including tests of normality (Shapiro–Wilk) and homogeneity of variance (Levene).

| **Trait** | **DF (Treatment/Error)** | **Mean Square (Treatment)** | **Mean Square (Error)** | **CV (%)** | **Shapiro–Wilk W** | **p (Shapiro)** | **Levene F** | **p (Levene)** |
| --- | --- | --- | --- | --- | --- | --- | --- | --- |
| Number of buds | 3 / 16 | 12.72 ** | 0.55 | 6.9 | 0.901 | 0.163 | 0.365 | 0.781 |
| Leaf length | 3 / 16 | 219.12 ** | 0.70 | 9.3 | 0.965 | 0.078 | 0.751 | 0.552 |
| Leaf width | 3 / 16 | 29.80 ** | 1.92 | 3.01 | 0.970 | 0.085 | 0.727 | 0.564 |
| Fresh weight | 3 / 16 | 115426.11 ** | 1630.59 | 3.7 | 0.968 | 0.088 | 0.333 | 0.802 |
| Dry weight | 3 / 16 | 4964 ** | 106.66 | 8.8 | 0.966 | 0.082 | 0.615 | 0.624 |

| Trait | DF  (Treatment/Error) | Mean Square (Treatment) | Mean Square (Error) | CV (%) | Shapiro–Wilk W | p (Shapiro) | Levene F | p (Levene) |
| --- | --- | --- | --- | --- | --- | --- | --- | --- |
| Number of buds | 2 / 6 | 6.47 ** | 0.33 | 6.9 | 0.918 | 0.179 | 0.599 | 0.565 |
| Leaf length | 2 / 6 | 27.80 ** | 0.53 | 9.3 | 0.907 | 0.123 | 0.696 | 0.518 |
| Leaf width | 2 / 6 | 19.4 ** | 0.67 | 3.01 | 0.962 | 0.721 | 1.474 | 0.268 |
| Fresh weight | 2 / 6 | 3019.47 ** | 2.87 | 3.7 | 0.953 | 0.575 | 1.127 | 0.356 |
| Dry weight | 2 / 6 | 26.47 ** | 0.10 | 8.8 | 0.940 | 0.379 | 0.791 | 0.476 |

Table 2. Analysis of variance of the effect of gamma irradiation doses on morphometric traits of seedless barberry (*Berberis vulgaris* L.) under in vitro condition, including tests of normality (Shapiro–Wilk) and homogeneity of variance (Levene).

**Significance is at the 1% probability level.
